# Efficacy of Remimazolam besylate for intravenous sedation in dental procedures for patients with cognitive disabilities: a prospective single-arm observational study

**DOI:** 10.1186/s12903-026-08424-1

**Published:** 2026-06-26

**Authors:** Giovanni Battista Grossi, Gregorio Menozzi, Matteo Pellegrini, Marco Carraro, Laura Rey, Angela Galeotti, Andrea Scribante, Anna Papaleo, Giuseppe Sofi

**Affiliations:** 1 Department of Biomedical, Surgical and Dental Sciences, University of Milan, Milan, 20122 Italy; 2 Maxillofacial Surgery and Dental Unit, Fondazione IRCCS Ca’ Granda Ospedale Maggiore Policlinico, University of Milan, Via Commenda 10, Milan, 20122 Italy; 3 Section of Dentistry, Department of Clinical, Surgical, Diagnostic and Pediatric Sciences, University of Pavia, Pavia, 27100 Italy; 4 Dentistry Unit, Management Innovations, Diagnostics and Clinical Pathways, Bambino Gesù Children’s Hospital, IRCCS, Rome, 00165 Italy; 5 U.N.-E.U. International Research Project on Human Health, Oral Health Section, Geneve, 1200 Switzerland; 6 Anesthesia and Intensive Care for Women and Children Unit, Fondazione IRCCS Ca’ Granda Ospedale Maggiore Policlinico, Milan, 20122 Italy

**Keywords:** Cognitive disability, Moderate sedation, Dental procedures, Intravenous sedation, Remimazolam, Special care dentistry.

## Abstract

**Objectives:**

To assess the efficacy and safety of intravenous remimazolam besylate for sedation during dental procedures in patients with cognitive disabilities, a group frequently managed under general anesthesia due to poor cooperation and increased perioperative risks.

**Materials and methods:**

In this single-center prospective single-arm observational study, 43 adult patients with cognitive or motor disabilities (ASA II–III) received outpatient dental care under intravenous remimazolam. Sedation was titrated to moderate levels according to EMA and IACSD guidelines. Outcomes included procedural success, Ramsay Sedation Scale, Post-Anesthetic Discharge Scoring System (PADSS), Modified Aldrete Score (MAS), vital signs, drug dosage, recovery, adverse events, and caregiver feedback.

**Results:**

All treatments were successfully completed without anesthesiologist intervention or flumazenil reversal. Mean onset of sedation was 3.8 ± 2.3 min, recovery time 47 ± 20.4 min, and time to discharge 72.5 ± 22.5 min. The mean total remimazolam dose was 14 ± 5.9 mg. Most patients reached Ramsay 4, while 9.7% remained ≤ 3. Vital parameters remained stable with no episodes of hypoxemia or airway compromise. At discharge, PADSS averaged 8.9 ± 1.0 and MAS 9.4 ± 0.6, confirming recovery despite motor or neurological limitations affecting PADSS scoring. Caregiver interviews indicated that 42% of patients slept during the day, 85% slept normally at night, 68% appeared calmer, and only 7% experienced minor adverse effects such as nausea or brief agitation.

**Conclusions:**

Remimazolam provided safe and effective intravenous sedation with rapid onset and reliable recovery. Combining PADSS with MAS improved discharge assessment in this patient population.

**Clinical relevance:**

Remimazolam deserves further investigation as a potential practical alternative to general anesthesia for dental procedures in patients with cognitive disabilities, with the potential to enable safe care, procedural success, and favorable recovery profiles.

**Trial registration:**

International Standard Randomised Controlled Trial Number (ISRCTN) registry, ISRCTN39322806. Retrospectively registered on 10 November 2025.

**Supplementary Information:**

The online version contains supplementary material available at https://doi.org/10.1186/s12903-026-08424-1.

## Introduction

 Dental treatment in patients with cognitive disabilities is often challenging due to impaired cooperation, communication difficulties, and behavioral dysregulation, frequently necessitating sedation to ensure safe and effective care [1–4]. Intravenous sedation represents a valuable alternative to general anesthesia, allowing improved patient management while reducing perioperative risks [5–7].

Remimazolam besylate is a short-acting benzodiazepine that has demonstrated rapid onset, favorable safety, and reliable recovery profiles in various procedural settings [8–11]. Preliminary evidence in dentistry suggests promising results, including high procedural success and minimal adverse events [12–15].

However, to date, no study has specifically evaluated remimazolam for intravenous sedation in patients with cognitive disabilities, a population characterized by increased clinical complexity and unpredictable responses to sedation [16, 17].

This prospective observational single-arm study therefore investigates the efficacy (primary endpoint: sedation success rate) and safety of remimazolam in this setting, aiming to provide preliminary evidence to support its use in special care dentistry.

## Materials and methods

### Design

This single-center, prospective, single-arm observational study was approved by the local ethics committee (Fondazione IRCCS Ca’ Granda Ospedale Maggiore Policlinico, Milan; Territorial Ethics Committee Lombardy 3; Trial ID 4996) and conducted in accordance with the Declaration of Helsinki (1964 and subsequent amendments).

The study was reported in accordance with the Strengthening the Reporting of Observational Studies in Epidemiology (STROBE) guidelines [18] (Supplementary Material 1). Although the present study was not designed as a randomized controlled trial, its reporting was aligned, where applicable, with the Consolidated Standards of Reporting Trials (CONSORT) recommendations [19] to enhance transparency and methodological clarity; the completed CONSORT checklist is provided as Supplementary Material 2. Adult patients with cognitive disabilities requiring dental procedures under intravenous sedation were consecutively enrolled after informed consent from legal guardians.

All procedures were performed in a hospital dental setting by trained clinicians, in accordance with the Intercollegiate Advisory Committee for Sedation in Dentistry (IACSD) standards [20].

### Sample size

In a previous clinical trial on remimazolam, the observed success rate of sedation without rescue medication was 100% [15]. For the present study in patients with disabilities, a conservative expected success rate of 95% was assumed, supported by previous evidence reporting comparably high success rates in outpatient oral and maxillofacial surgery [21]. This approach is consistent with methodological recommendations to avoid extreme proportions in sample size estimation [22]. The reference proportion was set at 80%, considered the minimum clinically acceptable success rate for routine use in vulnerable populations [23].

The sample size was calculated for a one-sided superiority design with α = 0.05 and 90% power, yielding 39 evaluable patients. Considering a 10% attrition rate, in line with rates reported in prospective clinical research [24], the final sample size was set at 43 patients. A one-sided test was chosen because the hypothesis of interest was that the true success rate would exceed the predefined clinically acceptable benchmark [25].

### Participants and setting

A total of 43 adult patients with cognitive disabilities were recruited between January and July 2025 at the Dental Sedation Service of the Maxillofacial Surgery and Dental Unit, Fondazione IRCCS Ca’ Granda Ospedale Maggiore Policlinico (Milan, Italy).

Inclusion criteria were age ≥ 18 years and the need for dental treatment under intravenous sedation. Exclusion criteria included contraindications to benzodiazepines, severe systemic comorbidities incompatible with moderate sedation, and pregnancy or breastfeeding. Written informed consent was obtained from legal guardians.

All procedures were conducted in a fully equipped hospital environment with continuous monitoring (pulse oximetry, non-invasive blood pressure, heart rate, respiratory rate, and capnography), in accordance with IACSD standards [26].

### Administration of Remimazolam besylate for dental procedural sedation

Remimazolam besylate (Byfavo^®^ 20 mg) was administered intravenously according to IACSD [26] and European Medicines Agency (EMA) [27] recommendations.

Initial dosing consisted of 7 mg in standard-risk patients or 2.5–5 mg in elderly or high-risk patients (ASA ≥ III), followed by maintenance doses of 1.25–2.5 mg at ≥ 2-minute intervals. Maximum cumulative doses were 33 mg or 17.5 mg, respectively.

Sedation failure was defined as failure to achieve the target sedation level after five administrations within 15 min. All sedations were performed by trained dental clinicians with staff skilled in airway management. Emergency equipment and flumazenil were available at all times [20].

### Outcome variables

The primary outcome was procedural success without the need for rescue sedatives. Secondary outcomes included sedation depth assessed by the Ramsay Sedation Scale [28], recovery evaluated using the Post-Anesthetic Discharge Scoring System (PADSS) [29] and the Modified Aldrete Score (MAS) [30], as well as vital parameters (SBP, DBP, MAP, HR, RR, SpO₂, EtCO₂), anesthesiologist intervention rate, amnesia, adverse events, intra- and postoperative pain, and caregiver-reported outcomes.

Detailed descriptions of the scoring systems are provided in Supplementary Material 3.

Discharge criteria included stable vital signs, PADSS ≥ 8, and MAS ≥ 9. In addition, recovery was clinically confirmed by the operator and caregiver, and discharge was permitted only in the presence of a responsible caregiver. In cases of delayed recovery (> 2 h), flumazenil administration was considered [27].

### Interventions

At baseline, demographic data, ASA classification, and medical history were recorded with caregiver input. Preoperative fasting followed the 2–4-6–8 rule [31].

On the day of treatment, monitoring was initiated before or immediately after induction in uncooperative patients. Local anesthesia was administered once adequate sedation (Ramsay score = 4) was achieved. Vital signs were continuously recorded throughout the procedure.

After treatment, patients were monitored until discharge criteria were met. Data collected included procedural success, duration of treatment, total remimazolam dose, onset and recovery times, and vital parameters.

Caregivers were contacted within 24 h post-procedure (T2) to assess postoperative effects, adverse events, and behavioral changes (Supplementary Material 4). The complete operating protocol is summarized in Table 1.

**Table 1:** Operating protocol designed for the study

**Appointment**	**Procedures**
Patient Selection and Recruitment (T-1)	- Patient selection conducted during the initial visit with caregivers/legal guardians. - Clinical data collected: age, sex, weight, height, BMI. - Medical history and current medications obtained from caregivers. - ASA classification recorded. - Complexity of the procedure: surgical/non-surgical; expected duration: short/long. - Vital parameters measured: SBP, DBP, MAP, HR, SpO₂ whenever patient cooperation allowed. - Written informed consent signed by legal guardian. - Inclusion/exclusion criteria verified before scheduling the procedure. - Pre-procedure instructions: adherence to the 2–4-6–8 fasting rule. - Notification to caregivers regarding the follow-up phone interview on the day after the procedure.
Day of the Dental Procedure (T0)	- Confirmation of medical history and fasting status with caregivers. - Peripheral venous access was established with an 18–22G cannula, depending on vein size. - Standard monitoring equipment applied (pulse oximetry, non-invasive blood pressure, ECG, RR, SpO₂, EtCO₂), whenever cooperation allowed; otherwise, completed after adequate sedation depth. - Remimazolam besylate (Byfavo® 20 mg) prepared and administered according to EMA and IACSD 2023 guidelines, with induction and maintenance dosing tailored to age, ASA, and weight. - Local anesthesia (plexus or mandibular block) performed only after achieving adequate sedation depth (Ramsay = 4). - Vital parameters recorded after each administration of remimazolam. - Anesthesiologist intervention requested if Ramsay scores ≥5 or complications occurred.
End of Procedure (T1)	- Post-procedure monitoring continued until stable vital signs achieved. - In case of delayed recovery (>2 h), flumazenil (0.2 mg) considered under anesthesiologist supervision. - Data recorded: sedation success rate, procedure duration, total remimazolam dose, onset time (from first injection to Ramsay 4/6), recovery time (to PADSS ≥8), anesthesiologist interventions, and vital parameters at discharge (SBP, DBP, MAP, HR, RR, SpO₂, EtCO₂). - Discharge permitted only with a PADSS ≥8 and MAS ≥9 and in the presence of a responsible caregiver. - Written and verbal post-sedation instructions provided to caregivers.
Telephone Interview (T2)	- Caregivers contacted within 24 h post-discharge. - Data collected on: (i) clinical effects of benzodiazepine administration, (ii) presence of intra- or postoperative pain, (iii) adverse effects, and (iv) general caregiver observations.

*Abbreviations*: *ASA* American Society of Anesthesiologists, *BMI* Body Mass Index, *DBP* Diastolic Blood Pressure, *ECG* Electrocardiography, *EtCO*₂ End-Tidal Carbon Dioxide, *HR* Heart Rate, *IACSD* Intercollegiate Advisory Committee for Sedation in Dentistry, *MAP* Mean Arterial Pressure, *MAS* Modified Aldrete Score, *PADSS* Post Anesthetic Discharge Scoring System, *RR* Respiratory Rate, *SBP* Systolic Blood Pressure, *SpO*₂ Peripheral

### Statistical methods

Descriptive statistics summarized the study outcomes. Continuous variables were expressed as means ± SD or medians (IQR), depending on Shapiro-Wilk normality testing; categorical variables were presented as counts and percentages. The primary outcome (success rate) was expressed as a percentage. Secondary outcomes were analyzed descriptively. Vital parameters across phases were compared using the Kruskal-Wallis test with Dunn’s post hoc correction. Recovery times were analyzed with the Kaplan–Meier estimator. Statistical significance was set at *p* < 0.05. Analyses were conducted using R (v4.4.0; R Foundation for Statistical Computing, Vienna, Austria). The study complied with 2020 IACSD standards [27].

## Results

### Demographic and clinical characteristics

The study included 43 patients (67.4% male), with a mean age of 40.3 ± 18.9 years (range: 18–83). Mean BMI was 24.0 ± 5.4 kg/m². Most patients were ASA III (83.7%), while 16.3% were ASA II (Table 2).

**Table 2:** Demographic, clinical, and anxiety characteristics of the study population

Sex (%)	Male = 67.40
	Female = 32.60
Age (years, mean ± SD; min-median-max)	40.28 ± 18.89; 18–34-83
Country of Birth (%)	Africa – Egypt = 2.33
	Europe – Italy = 93.02
	Europe – Bulgaria = 2.33
	Europe – Hungary = 2.33
Weight (Kg, mean ± SD; min-median-max)	67.11 ± 16.22; 29–64-87
Height (cm, mean ± SD; min-median-max)	167.10 ± 9.94; 140–168-185
BMI (mean ± SD; min-median-max)	24.02 ± 5.35; 11.30–23.51.30.51-33.50
ASA Classification (%)	ASA 1 = 0
	ASA 2 = 16.28
	ASA 3 = 83.72
	ASA 4 = 0

*Abbreviations*: *ASA* American Society of Anesthesiologists, *BMI* Body Mass Index, *Max* Maximum, *Min* Minimum, *SD* Standard Deviation

### Dental procedures and cognitive disabilities

**Fig. 1:**
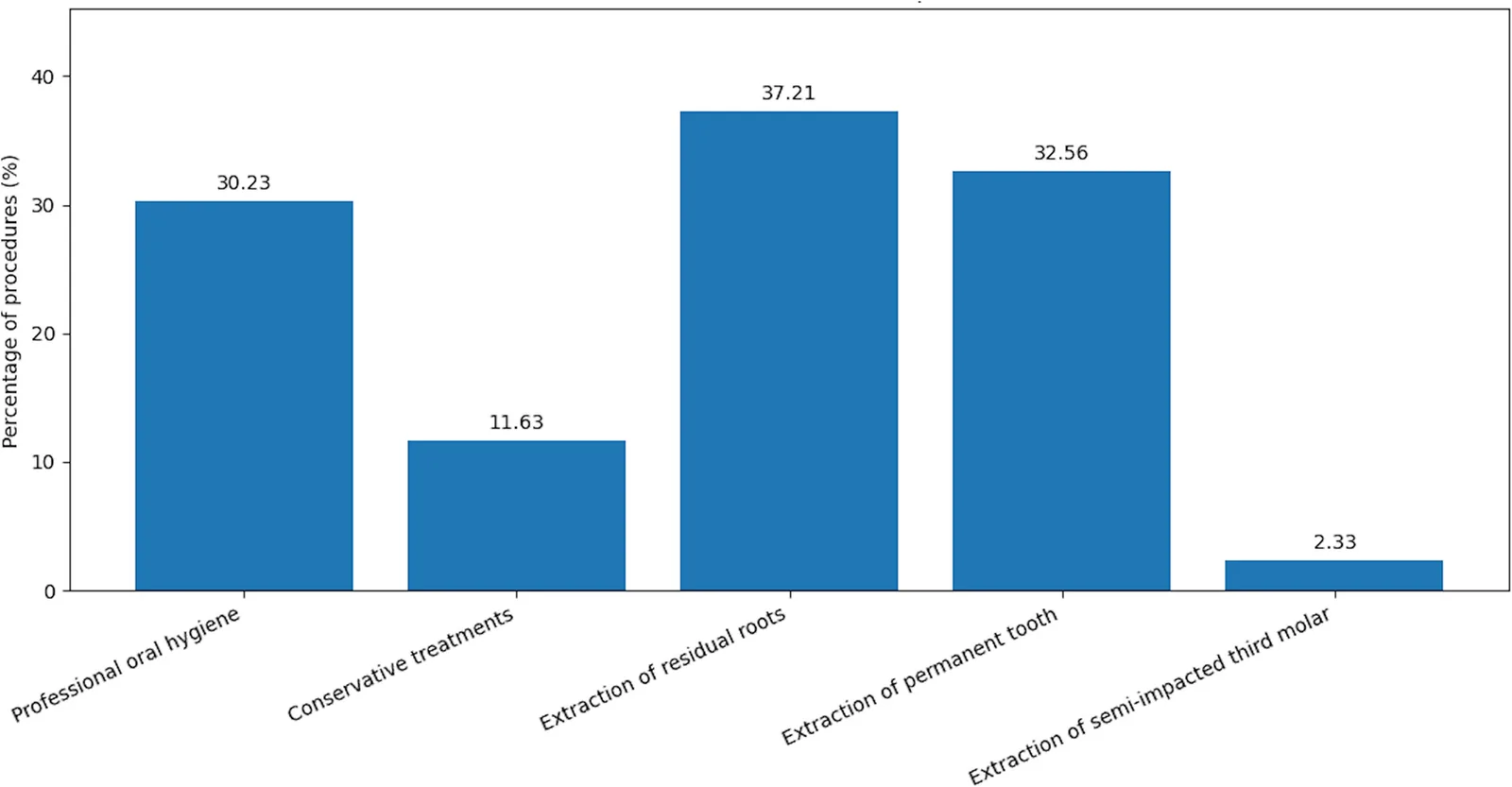
Distribution of scheduled dental procedures performed in the study population

Procedural distribution is summarized in Fig. 1. The study included a range of dental procedures, namely professional oral hygiene (30.23%), conservative treatments (11.63%), extraction of residual roots (37.21%), extraction of permanent teeth (32.56%), and extraction of semi-impacted third molars (2.33%). Extractions represented the most frequent interventions overall.

**Fig. 2:**
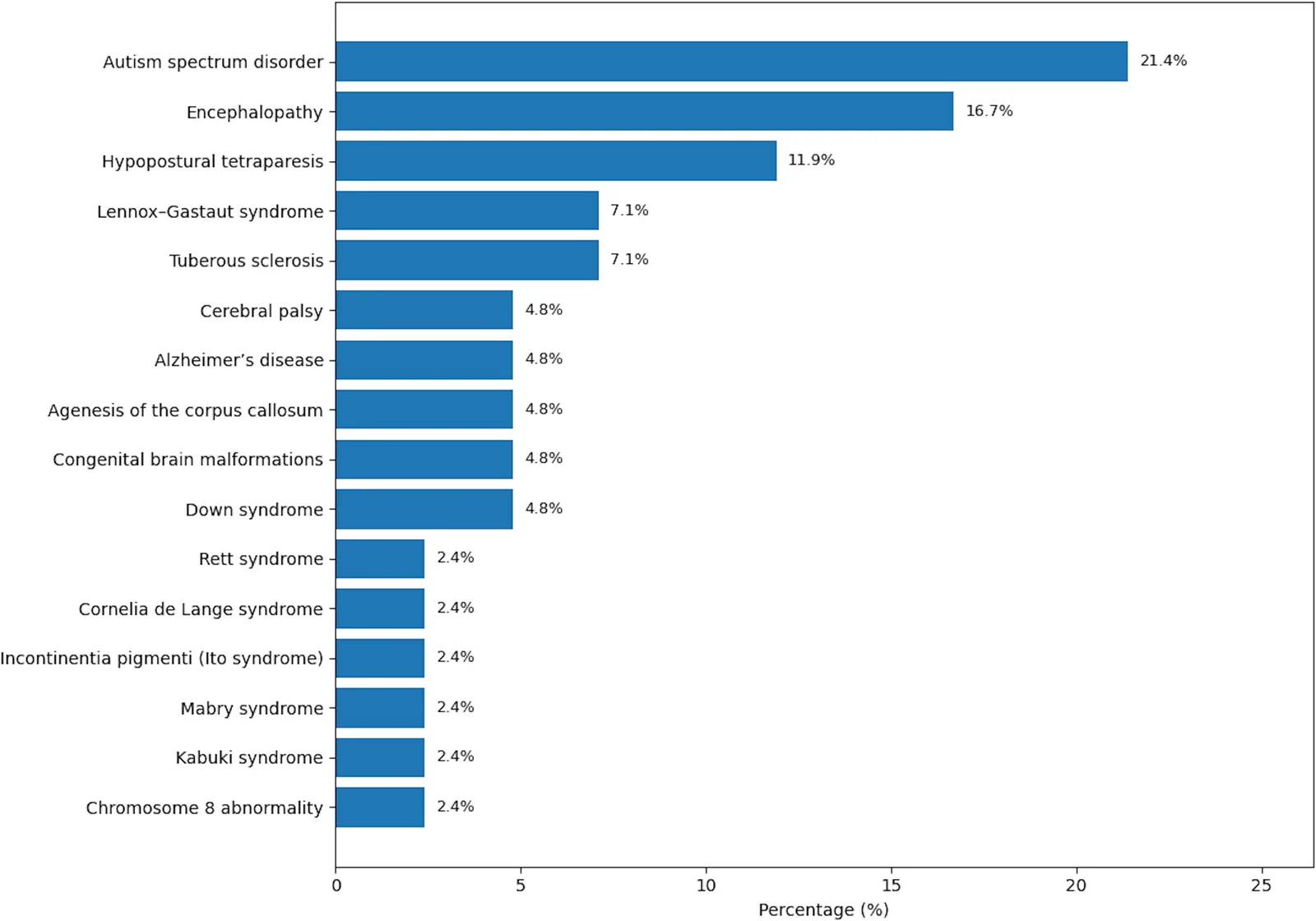
Bar chart showing the relative frequency of different cognitive disabilities in the study population. Autism spectrum disorder and encephalopathy were the most prevalent conditions, followed by hypopostural tetraparesis and Lennox–Gastaut syndrome, while several rare syndromes were observed with lower frequencies

Autism spectrum disorder was the most prevalent condition (21.4%), followed by encephalopathy (16.7%) and hypopostural tetraparesis (11.9%). Other conditions were less frequent (Fig. 2).

### Medical conditions and medications

Epilepsy was the most common comorbidity (69.8%), followed by hypertension (15.2%), obesity (14.0%), and iron-deficiency anemia (11.6%).

Anticonvulsants were the most frequently prescribed drugs (32.6%), followed by proton pump inhibitors and benzodiazepines (20.9% each). Further details are provided in Supplementary Material 5.

### Vital signs during procedural phases

Vital parameters remained stable across procedural phases (Table 3). SBP, DBP, MAP, SpO₂, and EtCO₂ showed no clinically relevant variations.

**Table 3:** Vital signs across different phases of the procedure

		SBP (mmHg)	DBP (mmHg)	MAP (mmHg)	HR (bpm)	RR (breaths/min)	SpO_2_ (%)	EtCO_2_ (mmHg)
Recruitment Phase (mean ± SD; min-median-max)		125.6 ± 15.9; 101–128–169	74.7 ± 12.8; 41–78–98	92.6 ± 12.2; 63–93–124	81.9 ± 18.8; 50–80–136	/	97.2 ± 1.9; 90–98–100	/
Preoperative Phase (mean ± SD; min-median-max)		128.1 ± 21.6; 92–124–172	75.3 ± 10.8; 55–75–97	99.3 ± 19.0; 73–97–149	79.7 ± 19.8; 52–74–146	20.0 ± 5.5; 12–19.5–39	97.2 ± 2.2; 90–98–100	34.8 ± 4.0; 25–34.5–43
Induction Phase (mean ± SD; min-median-max)	Induction I	132.3 ± 21.9; 93–131–182	74.8 ± 11.6; 51–73–99	97.8 ± 18.5; 72–93–153	82.4 ± 15.5; 53–79–119	18.3 ± 5.4; 5–18–32	96.7 ± 3.1; 87–97–100	34.5 ± 5.8; 13–35–44
	Induction II	135.0 ± 22.0; 100–131.5–198	76.0 ± 10.2; 55–74–97	99.0 ± 15.6; 63–94–129	90.3 ± 25.4; 18–89.5–145	20.6 ± 9.5; 5–18–54	96.2 ± 2.6; 90–96–100	35.1 ± 4.5; 20–36–42
	Induction III	132.6 ± 16.6; 110–127–163	78.6 ± 10.6; 58–77–91	98.0 ± 16.6; 75–93–122	90.9 ± 18.6; 74–87–131	18.2 ± 3.5; 13–18.5–24	96.8 ± 2.4; 93–96–100	34.3 ± 6.7; 20–36–43
	Induction IV	138.8 ± 15.1; 120–139–157	77.3 ± 4.6; 72–77–83	99.0 ± 6.5; 93–97.5–108	113.3 ± 21.3; 86–115–137	22.3 ± 4.2; 18–21.5–28	98.5 ± 1.7; 96–99–100	34.3 ± 9.7; 20–37.5–42
	Induction V	129.5 ± 13.4; 120–129.5–139	79.5 ± 12.0; 71–79.5–88	98.0 ± 5.7; 94–98–102	110.0 ± 29.7; 89–110–131	18.5 ± 6.4; 14–18.5–23	98.5 ± 2.1; 97–98.5–100	26.0 ± 11.3; 18–26–34
Maintenance Phase (mean ± SD; min-median-max)	Maintenance I	134.5 ± 21.0; 105–131–194	79.0 ± 15.9; 55–75–133	102.3 ± 17.6; 77.3–97–148	89.1 ± 18.7; 57–87.5–150	20.1 ± 5.6; 11–19.5–36	96.5 ± 2.9; 87–97–100	35.0 ± 3.6; 26–35–44
	Maintenance II	131.2 ± 15.9; 108–129.5–180	72.8 ± 10.9; 52–72.5–97	95.6 ± 14.4; 67–91.5–125	90.8 ± 17.8; 57–88–143	19.9 ± 6.1; 9–20–35	96.6 ± 2.8; 88–97–100	35.4 ± 4.8; 21–36–46
	Maintenance III	129.2 ± 10.7; 118–127–160	76.4 ± 10.9; 62–73.5–99	93.7 ± 10.7; 82–90–115	98.5 ± 17.2; 76–90–131	19.7 ± 7.1; 12–18–38	96.6 ± 2.1; 93–97–100	34.7 ± 3.6; 26–35.5–39
	Maintenance IV	130.0 ± 11.4; 112–128–145	75.7 ± 13.6; 65–72–114	99.1 ± 17.5; 82–94–136	106.0 ± 25.5; 76–99–162	22.7 ± 5.9; 14–21–35	96.7 ± 2.6; 92–97–100	35.5 ± 3.1; 29–37–38
	Maintenance V	128.2 ± 12.3; 117–125.5–152	64.8 ± 8.2; 54–66.5–76	86.5 ± 5.5; 77–88.5–92	111.7 ± 22.7; 93–101.5–148	20.2 ± 3.4; 18–19–27	97.3 ± 1.6; 96–97–100	35.2 ± 3.2; 31–35.5–39
	Maintenance VI	124.2 ± 16.0; 108–122–144	70.2 ± 16.8; 51–76–91	88.0 ± 13.5; 71–88–108	100.3 ± 23.9; 84–91–135	19.8 ± 0.8; 19–20–21	96.8 ± 1.3; 96–96–99	34.6 ± 3.0; 31–36–38
	Maintenance VII	126.0 ± 9.6; 119–122–137	65.7 ± 8.6; 58–64–75	87.3 ± 6.7; 83–84–95	105.0 ± 28.4; 83–95–137	17.0 ± 4.6; 12–18–21	98.3 ± 1.5; 97–98–100	34.3 ± 3.1; 31–35–37
	Maintenance VIII	123.0 ± 18.4; 110–123–136	52.0 ± 1.4; 51–52–53	75.5 ± 6.4; 71–75.5–80	116.5 ± 27.6; 97–116.5–136	19.0 ± 1.4; 18–19–20	96.0; 96–96–96	33.5 ± 3.5; 31–33.5–36
	Maintenance IX	125.5 ± 4.9; 122–125.5–129	61.0 ± 4.2; 58–61–64	84.5 ± 2.1; 83–84.5–86	117.0 ± 31.1; 95–117–139	14.5 ± 4.9; 11–14.5–18	97.5 ± 0.7; 97–97.5–98	34.5 ± 4.9; 31–34.5–38
	Maintenance X	110.0; 110–110–110	51.0; 51–51–51	71.0; 71–71–71	97.0; 97–97–97	20.0; 20–20–20	96.0; 96–96–96	31.0; 31–31–31
End of Procedure (mean ± SD; min-median-max)		129.7 ± 20.0; 92–128–189	72.9 ± 13.5; 46–74–105	97.2 ± 15.2; 70–96–139	87.2 ± 17.3; 51–84–138	18.4 ± 5.4; 7–17–32	96.6 ± 3.0; 87–97–100	34.1 ± 6.2; 12–35–45
Discharge (mean ± SD; min-median-max)		127.5 ± 18.8; 90–128–174	76.3 ± 12.6; 55–74–122	95.7 ± 14.2; 71–91–139	79.6 ± 18.9; 55–74–137	/	97.3 ± 2.1; 93–97–100	/

*Abbreviations*: *bpm* beats per minute, *DBP* Diastolic Blood Pressure, *EtCO*_2_ End-Tidal Carbon Dioxide, *HR* Heart Rate, *MAP* Mean Arterial Pressure, *mmHg* millimeters of mercury, *Max* Maximum, *Min* Minimum, *RR* Respiratory Rate, *SBP* Systolic Blood Pressure, *SD* Standard Deviation, *SpO*_2_ Oxygen Saturation

Heart rate showed significant differences across phases (Kruskal–Wallis test), with a decrease between the preoperative phase and maintenance IV (*p* = 0.0421) and an increase between maintenance IV and discharge (*p* = 0.0366) (Table 4).

**Table 4:** Vital signs and ramsay dunn’s multiple comparisons test

**Outcomes**	**Multiple comparisons**	**Mean rank difference**	**p-value**
HR	Preoperative Phase vs Maintenance IV	−132,1	0.0421
	Maintenance IV vs Discharge	131,2	0.0366
Ramsay	Induction I vs End of Procedure	−49.23	0.023

*Abbreviations*: *HR* Heart Rate

### Sedation profile and drug administration

Initial remimazolam dosing varied (2.5–7 mg), while subsequent administrations were predominantly 2.5 mg (Table 5).

**Table 5:** Remimazolam dosage, Ramsay, PADSS and MAS scores by phase

		**Remimazolam (%)**	**Ramsay score (%)**	**PADSS score (mean ± SD; min-median-max)**	**MAS score (mean ± SD; min-median-max)**
Preoperative Phase		/	/	/	/
Induction Phase	Induction I	2.5 mg = 22.73 5.0 mg = 45.45 7.0 mg = 31.82	1 = 6.25 2 = 0 3 = 0 4 = 93.75 5 = 0 6 = 0	/	/
	Induction II	1.25 mg = 0 2.5 mg = 100.00	1 = 7.89 2 = 0 3 = 0 4 = 92.11 5 = 0 6 = 0	/	
	Induction III	1.25 mg = 0 2.5 mg = 100.00	1 = 11.76 2 = 0 3 = 0 4 = 88.24 5 = 0 6 = 0	/	
	Induction IV	1.25 mg = 0 2.5 mg = 100.00	1 = 9.52 2 = 0 3 = 0 4 = 90.48 5 = 0 6 = 0	/	
	Induction V	1.25 mg = 0 2.5 mg = 100.00	1 = 8.70 2 = 0 3 = 0 4 = 91.30 5 = 0 6 = 0	/	
Maintenance Phase	Maintenance I	1.25 mg = 2.44 2.5 mg = 97.56 5 mg = 0	1 = 10.53 2 = 0 3 = 0 4 = 89.47 5 = 0 6 = 0	/	
	Maintenance II	1.25 mg = 2.63 2.5 mg = 97.37 5 mg = 0	1 = 7.14 2 = 0 3 = 0 4 = 92.86 5 = 0 6 = 0	/	
	Maintenance III	1.25 mg = 0 2.5 mg = 100.00 5 mg = 0	1 = 12.50 2 = 0 3 = 0 4 = 87.50 5 = 0 6 = 0	/	
	Maintenance IV	1.25 mg = 0 2.5 mg = 100.00 5 mg = 0	1 = 4.76 2 = 0 3 = 0 4 = 95.24 5 = 0 6 = 0	/	
	Maintenance V	1.25 mg = 0 2.5 mg = 100.00 5 mg = 0	1 = 14.29 2 = 0 3 = 0 4 = 85.71 5 = 0 6 = 0	/	
	Maintenance VI	1.25 mg = 0 2.5 mg = 100.00 5 mg = 0	1 = 9.09 2 = 0 3 = 0 4 = 90.91 5 = 0 6 = 0	/	
	Maintenance VII	1.25 mg = 0 2.5 mg = 100.00 5 mg = 0	1 = 8.33 2 = 0 3 = 0 4 = 91.67 5 = 0 6 = 0	/	
	Maintenance VIII	1.25 mg = 0 2.5 mg = 100.00 5 mg = 0	1 = 11.11 2 = 0 3 = 0 4 = 88.89 5 = 0 6 = 0	/	
	Maintenance IX	1.25 mg = 0 2.5 mg = 100.00 5 mg = 0	1 = 9.09 2 = 0 3 = 0 4 = 90.91 5 = 0 6 = 0	/	
	Maintenance X	1.25 mg = 0 2.5 mg = 100.00 5 mg = 0	1 = 10.00 2 = 0 3 = 0 4 = 90.00 5 = 0 6 = 0	/	
End of Procedure		/	1 = 13.64 2 = 0 3 = 0 4 = 86.36 5 = 0 6 = 0	/	/
Discharge		/	/	8.86 ± 0.97; 8-8-10	9.4 ± 0.6 (9-9-10)

*Abbreviations*: *MAS* Modified Aldrete Score, *Max* Maximum, *Min* Minimum, *PADDS* Post Anesthesia Discharge Scoring System, *SD* Standard Deviation, *VAS-T* Visual Analog Scale for Tranquility

Ramsay sedation scores were predominantly level 4 across all phases (≈ 85–95%). A significant difference across phases was observed, with lower sedation levels at the end of the procedure compared to induction (*p* = 0.023).

At discharge, PADSS was 8.86 ± 0.97 and MAS was 9.4 ± 0.6 (Table 5).

### Procedural outcomes and recovery

All procedures were completed successfully without anesthesiologist intervention or flumazenil use (Table 6).

**Table 6:** Timing, total remimazolam administration, and outcomes

Success of the procedure (%)	100.00
Total mg Remimazolam Administered (mean ± SD; min-median-max)	14.0 ± 5.9; 4.0–12.5.0.5-32.0
Total Procedure Time (minutes, mean ± SD; min-median-max)	12.7 ± 7.0; 3.0–12.0.0.0-44.0
Time from start to discharge (minutes, mean ± SD; min-median-max)	72.5 ± 22.5; 30.0–71.0.0.0-135.0
Onset Time (minutes, mean ± SD; min-median-max)	3.8 ± 2.3; 2.0–3.0.0.0-10.0
Recovery Time (minutes, mean ± SD; min-median-max)	47.4 ± 20.4; 10.0–47.0.0.0-79.0
Ramsay Score ≤3 during the entire procedure (%)	9.70
Operator observed patient sleeping during the procedure (%)	90.30
Operator observed signs of pain during the procedure (e.g., agitation, crying, grimacing, self-protective movements) (%)	67.40
Anesthetist Intervention (Ramsay ≥5) (%)	0
Flumazenil Administration (%)	0

*Abbreviations*: *Max* Maximum, *Min* Minimum, *SD* Standard Deviation

Mean remimazolam dose was 14.0 ± 5.9 mg. Mean procedure time was 12.7 ± 7.0 min. Sedation onset occurred at 3.8 ± 2.3 min, with recovery at 47.4 ± 20.4 min and discharge at 72.5 ± 22.5 min.

Most patients reached Ramsay level 4, while 9.7% remained ≤ 3. Patients appeared asleep in 90.3% of cases, while signs of discomfort were observed in 67.4%.

### Postoperative outcomes

At 24-hour follow-up, 42% of patients slept during the day after the procedure and 85% slept normally at night. Most patients appeared calmer (68%), while 30% reported postoperative pain.

Adverse events occurred in 7% of cases and included mild nausea (3%), vomiting (2%), and transient agitation (2%). Further details are reported in Table 7.

**Table 7:** Telephone interview results performed the day after the procedure

Effects of the Administered Benzodiazepine			
* The patient slept at home after the procedure (%)*	*The patient slept the night after the procedure (%)*		*After the procedure, the patient appeared (%)*
42.00	As usual = 85.00 Better than usual = 5.00 Worse than usual = 10.00		Calmer than usual = 68.00 As usual = 29.00 More agitated than usual = 3.00
Postoperative Pain			
* The patient showed signs of pain after the procedure (%)*			
30.00			
Adverse Effects			
* The patient perceived symptoms other than drowsiness or calmness (%)*		*Reported symptoms*	
7.00		Nausea = 3.00 Vomiting = 2.00 Transient agitation = 2.00	
General Caregiver Observations			
* How the patient spent the hours following the procedure (%)*	*The patient required special attention (%)*		*If yes, which ones (%)*
Calm = 55.00 Drowsy = 25.00 Normal = 18.00 Agitated = 2.00	12.00		Closer monitoring = 8.00 Help with feeding/hydration = 4.00

### Discharge profile

**Fig. 3:**
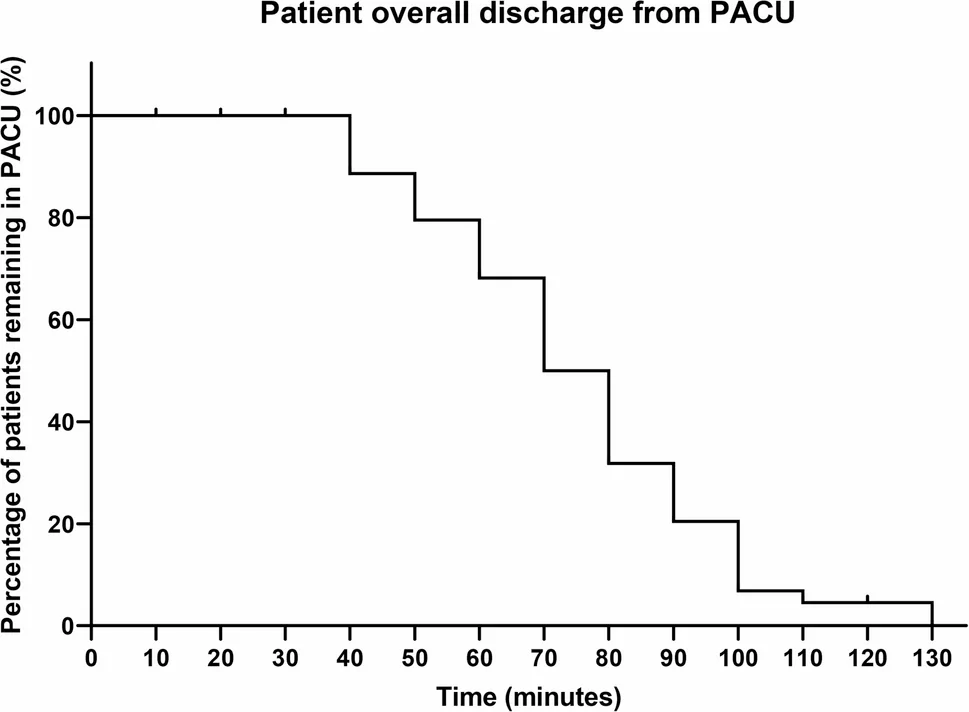
Kaplan–Meier curve illustrating the discharge timeline of 43 patients undergoing dental procedures under intravenous sedation with remimazolam. All 43 patients reached the event (discharge); no censored observations were present

The Kaplan–Meier analysis (Fig. 3) showed that approximately 50% of patients were discharged within 80 min, and all patients were discharged within 130 min.

## Discussion

This prospective study is, to our knowledge, the first to evaluate intravenous remimazolam sedation in dental patients with cognitive disabilities. The 100% procedural success rate confirms the feasibility of this approach in a high-risk population and is consistent with previous findings in cooperative adults undergoing dental procedures [12, 13, 32]. These results also align with broader clinical experiences supporting the reliability of remimazolam in dental settings [15, 33].

Sedation onset (3.8 min), recovery (47 min), and discharge times were consistent with previous reports [10, 15, 33–35]. Slightly prolonged recovery in our cohort may be related to the high proportion of ASA III patients and the frequent use of concomitant medications, particularly anticonvulsants and psychotropic drugs.

Procedural duration and overall time to discharge were comparable with previous studies in outpatient dental sedation [12, 32, 34, 36], supporting the suitability of remimazolam for short procedures. Its pharmacokinetic profile and minimal cardiorespiratory impact make it particularly relevant in fragile patients, where general anesthesia has traditionally been required [11, 15, 37].

Vital parameters remained stable throughout all phases, with no hypoxemia or airway compromise. These findings are consistent with previous evidence demonstrating favorable hemodynamic and respiratory safety profiles reported for remimazolam [34, 35, 38, 39].

Adequate sedation was achieved in most patients, with Ramsay level 4 predominating. However, behavioral signs of discomfort were frequently observed despite apparent adequate sedation. This discrepancy likely reflects the limitations of clinical sedation assessment in patients with cognitive disabilities, where non-verbal behaviors may not reliably correlate with nociception or sedation depth [12, 40, 41].

A relevant finding concerns recovery assessment. PADSS scores were frequently limited by impaired ambulation and responsiveness, potentially underestimating recovery in patients with severe neurological or motor impairment. Similar limitations have been reported in perioperative settings [42]. In contrast, MAS scores were consistently higher and more homogeneous, reflecting physiological recovery independently of motor function. These findings are consistent with previous observations suggesting that MAS may provide a more reliable assessment of recovery in vulnerable populations [30], and support its use in cognitively impaired dental patients.

Postoperative outcomes were favorable, with preserved sleep patterns, low rates of adverse events (7%), and generally positive caregiver-reported recovery. These findings are consistent with previous studies reporting rapid recovery and good tolerability of remimazolam [12, 34, 41, 43].

Overall, these results support the potential role of remimazolam in the management of patients with cognitive disabilities, enabling safe outpatient dental care. Recent studies have similarly highlighted its advantages in vulnerable populations, including shorter recovery times and improved perioperative efficiency [15, 43].

The absence of anesthesiologist intervention or flumazenil use in this study further supports the safety profile of remimazolam, consistent with previous reports [11, 39, 44].

This study has limitations. Its single-center design and limited sample size may restrict generalizability. The lack of a control group prevents direct comparison with other sedatives. In addition, sedation depth assessment relied on clinical scales that may be less reliable in non-verbal patients, and postoperative outcomes were based on caregiver reports. Follow-up was limited to the immediate postoperative period. Furthermore, the study population showed an imbalance in gender distribution and limited ethnic diversity, which may further restrict the generalizability of the findings. The predominance of male patients may reflect the underlying epidemiology of certain neurodevelopmental disorders, such as autism spectrum disorder, which are more frequently diagnosed in males, as well as potential referral patterns in this clinical setting [45, 46]. Although no clear sex-related differences in the efficacy or safety of remimazolam have been established, this imbalance should be considered when interpreting the results.

Despite these limitations, our findings support remimazolam as a safe and effective option for intravenous sedation in dental patients with cognitive disabilities. Further multicenter randomized controlled trials are needed to confirm these results and to compare remimazolam with established sedative agents.

## Conclusions

Remimazolam provided safe and effective intravenous sedation for dental treatment in patients with cognitive disabilities, with high procedural success and rapid recovery. However, the single-center design, small sample size, and absence of a control group limit the generalizability of these findings. Larger randomized controlled trials are required to confirm these results and to compare remimazolam with established sedative agents.

## Supplementary Information

Supplementary Material 1.

Supplementary Material 2.

Supplementary Material 3.

Supplementary Material 4.

Supplementary Material 5.

## Data Availability

No datasets were generated or analysed during the current study.
