# Supplementary material for: Efficacy of Remimazolam besylate for intravenous sedation in dental procedures for patients with cognitive disabilities: a prospective single-arm observational study
Source: BMC Oral Health. 2026 Jun 26;26:1704. doi: 10.1186/s12903-026-08424-1 (PMC13563919; doi:10.1186/s12903-026-08424-1)
Supplement: Supplementary file 2 [file 12903_2026_8424_MOESM2_ESM.docx]

**Supplementary Table 6.** Structured Telephone Interview Post-Procedure.

| **Category** | **Questions** | **Responses** |
| --- | --- | --- |
| **Effects of the administered benzodiazepine** | Did the patient sleep at home after the procedure? | • Yes • No |
|  | During the night following the procedure, the patient slept: | • As usual • Better than usual • Worse than usual |
|  | After the procedure, the patient appeared: | • Calmer than usual • As usual • More agitated than usual |
| **Postoperative pain** | After the procedure, did the patient show signs of pain (e.g., agitation, crying, grimacing, self-protective gestures)? | • Yes • No |
| **Adverse effects** | Did you notice any symptoms other than drowsiness or calmness after the procedure (e.g., nausea, vomiting, agitation, respiratory difficulties)? | • Yes • No |
|  | If yes, which ones? |  |
| **General caregiver observations** | How did the patient spend the hours following the procedure? | • Calm • Agitated • Drowsy • Normal |
|  | Did the patient require special attention compared to usual? | • Yes • No |
|  | If yes, which ones? |  |
