# Supplementary material for: Efficacy of Remimazolam besylate for intravenous sedation in dental procedures for patients with cognitive disabilities: a prospective single-arm observational study
Source: BMC Oral Health. 2026 Jun 26;26:1704. doi: 10.1186/s12903-026-08424-1 (PMC13563919; doi:10.1186/s12903-026-08424-1)
Supplement: Supplementary file 4 [file 12903_2026_8424_MOESM4_ESM.docx]

**Supplementary Table 7.** Prevalence of Diseases in the Study Population.

| **Cardiovascular Diseases (%)** | |
| --- | --- |
| Hypertension | 15.2 |
| Ischemic heart disease | 4.3 |
| Heart failure | 2.2 |
| Right bundle branch block | 2.2 |
| Dysphagic extrapyramidal syndrome | 2.2 |
| Ventriculoperitoneal shunt in secondary hydrocephalus due to thalamic hemorrhage | 2.2 |
| **Metabolic and Endocrine Diseases (%)** | |
| Type I diabetes mellitus | 4.7 |
| Type II diabetes mellitus | 2.3 |
| Class 1 obesity | 14.0 |
| Hypothyroidism | 4.7 |
| Hyperthyroidism | 2.3 |
| **Neurological Diseases (%)** | |
| Epilepsy | 69.8 |
| Stroke | 7.0 |
| Hemiparesis | 7.0 |
| Schizoaffective disorder | 2.3 |
| Dysphagia | 4.7 |
| Dysphasia | 4.7 |
| **Respiratory Diseases (%)** | |
| Asthma | 2.3 |
| Mild-moderate exertional dyspnea | 2.3 |
| OSAS | 4.7 |
| **Hematological and Coagulation Disorders (%)** | |
| Iron-deficiency anemia | 11.6 |
| Hemophilia | 2.3 |
| **Gastroenterological Diseases (%)** | |
| Diverticulosis | 2.3 |
| **Oncological Diseases (%)** | |
| Hemangioma | 4.7 |
| Meningioma | 2.3 |
| Rhabdomyoma | 4.7 |
| **Ophthalmological Diseases (%)** | |
| Glaucoma | 4.7 |
| Hemianopsia | 7.0 |
| Blindness | 4.7 |
| **Musculoskeletal Diseases (%)** | |
| Scoliosis | 2.3 |
| **Allergies (%)** | |
| Iron-Nickel | 2.3 |
| Amoxicillin + clavulanic acid | 2.3 |
| Ampicillin | 2.3 |
| Local anesthetics | 2.3 |
| Scopolamine butylbromide | 2.3 |
| Amiodarone | 2.3 |
| Lamotrigine | 2.3 |
| Acetylsalicylic acid | 4.7 |

Abbreviations: OSAS, Obstructive Sleep Apnea Syndrome.

**Supplementary Table 8.** Distribution of Medication in the Study Population.

| **Antihistamines (%)** | 2.3 |
| --- | --- |
| **Oral antidiabetics (%)** | 9.3 |
| **Diuretic (%)** | 9.3 |
| **Laxative (%)** | 4.7 |
| **Antipsychotics (%)** | 14.0 |
| **Anticonvulsants (%)** | 32.6 |
| **Benzodiazepines (%)** | 20.9 |
| **ACE inhibitors (%)** | 7.0 |
| **Calcium channel blockers (%)** | 2.3 |
| **Angiotensin II receptor antagonists (%)** | 11.6 |
| **Beta blockers (%)** | 7.0 |
| **Triptans (%)** | 2.3 |
| **Thyroid disease medications (%)** | 4.7 |
| **Sodium alginate and sodium bicarbonate (%)** | 2.3 |
| **Proton pump inhibitors (%)** | 20.9 |
| **Lipid-lowering agents (%)** | 11.6 |
| **Antidepressants (%)** | 14.0 |
| **Cannabinoids (%)** | 2.3 |
| **Anticoagulants (%)** | 2.3 |
| **Antiplatelets (%)** | 9.3 |
| **Alpha blockers (%)** | 2.3 |
| **Hormonal drugs (%)** | 2.3 |
| **Vitamin D (%)** | 2.3 |
| **Bronchodilator (%)** | 4.7 |
| **Immunosuppressants (%)** | 2.3 |

Abbreviations: ACE, Angiotensin-Converting Enzyme.
