# Supplementary material for: Efficacy of Remimazolam besylate for intravenous sedation in dental procedures for patients with cognitive disabilities: a prospective single-arm observational study
Source: BMC Oral Health. 2026 Jun 26;26:1704. doi: 10.1186/s12903-026-08424-1 (PMC13563919; doi:10.1186/s12903-026-08424-1)
Supplement: Supplementary file 5 [file 12903_2026_8424_MOESM5_ESM.docx]

**Supplementary Table 3.** Ramsay Sedation Scale.

| **Clinical Score** | **Patient Characteristics** |
| --- | --- |
| 1 | Awake; agitated or restless or both |
| 2 | Awake; cooperative, oriented, and tranquil |
| 3 | Awake but responds to commands only |
| 4 | Asleep; brisk response to light glabellar tap or loud auditory stimulus |
| 5 | Asleep; sluggish response to light glabellar tap or loud auditory stimulus |
| 6 | Asleep; no response to glabellar tap or loud auditory stimulus |

Ramsay MA, Savage TM, Simpson B, et al: Controlled sedation with alphaxalone-alphadolone. British Medical Journal 22;2(5920):656–659. doi: 10.1136/bmj.2.5920.656

**Supplementary Table 4.** Modified Post Anesthetic Discharge Scoring System (PADSS).

| **Domain** | **Score 2** | **Score 1** | **Score 0** |
| --- | --- | --- | --- |
| Vital Signs | Within 20% of preoperative baseline | Between 20-40% of preoperative baseline | Greater than 40% from preoperative baseline |
| Activity and Ambulation | Ambulates steadily without dizziness | Requires assistance | Unable to ambulate |
| Nausea and Vomiting | Minimal or absent | Moderate nausea/vomiting controlled | Severe nausea/vomiting |
| Pain | Minimal or controlled with oral medications | Moderate pain controlled with IV/IM medications | Severe pain |
| Surgical Bleeding | Minimal bleeding | Moderate bleeding (requires dressing change) | Severe bleeding (requires intervention) |

Palumbo P, Tellan G, Perotti B, Pacilè MA, Vietri F, Illuminati G. Modified PADSS (Post Anaesthetic Discharge Scoring System) for monitoring outpatients discharge. Ann Ital Chir. 2013 Nov-Dec;84(6):661-5.

**Supplementary Table 5.** Modified Aldrete Score (MAS).

| **Domain** | **Score 2** | **Score 1** | **Score 0** |
| --- | --- | --- | --- |
| Activity | Able to move 4 extremities voluntarily or on command | Able to move 2 extremities voluntarily or on command | Unable to move extremities |
| Respiration | Able to breathe deeply and cough freely | Dyspnea or limited, irregular breathing | Apnea |
| Circulation | Blood pressure ±20 mmHg of pre-anesthetic level | Blood pressure ±20–50 mmHg of pre-anesthetic level | Blood pressure ±50 mmHg of pre-anesthetic level |
| Consciousness | Fully awake | Arousable on calling | Not responding |
| Oxygenation | SpO₂ > 90% on room air | Needs supplemental O₂ to maintain SpO₂ > 90% | SpO₂ < 90% even with supplemental oxygen |

Deshmukh PP, Chakole V (2024) Post-Anesthesia Recovery: A Comprehensive Review of Sampe, Modified Aldrete, and White Scoring Systems. Cureus 16(10):e70935.
